# 8p22 *MTUS1* Gene Product ATIP3 Is a Novel Anti-Mitotic Protein Underexpressed in Invasive Breast Carcinoma of Poor Prognosis

**DOI:** 10.1371/journal.pone.0007239

**Published:** 2009-10-01

**Authors:** Sylvie Rodrigues-Ferreira, Anne Di Tommaso, Ariane Dimitrov, Sylvie Cazaubon, Nadège Gruel, Hélène Colasson, André Nicolas, Nathalie Chaverot, Vincent Molinié, Fabien Reyal, Brigitte Sigal-Zafrani, Benoit Terris, Olivier Delattre, François Radvanyi, Franck Perez, Anne Vincent-Salomon, Clara Nahmias

**Affiliations:** 1 Institut Cochin, Université Paris Descartes, Inserm U567, CNRS UMR8104, Paris, France; 2 CNRS UMR144, Institut Curie, Paris, France; 3 Inserm U830, Institut Curie, Paris, France; 4 Translational Research Department, Institut Curie, Paris, France; 5 Pathology Department, Hopital Curie, Paris, France; 6 Pathology Department, Hopital St. Joseph, Paris, France; 7 Pathology Department, Hopital Cochin, Paris, France; Roswell Park Cancer Institute, United States of America

## Abstract

**Background:**

Breast cancer is a heterogeneous disease that is not totally eradicated by current therapies. The classification of breast tumors into distinct molecular subtypes by gene profiling and immunodetection of surrogate markers has proven useful for tumor prognosis and prediction of effective targeted treatments. The challenge now is to identify molecular biomarkers that may be of functional relevance for personalized therapy of breast tumors with poor outcome that do not respond to available treatments. The Mitochondrial Tumor Suppressor (*MTUS1*) gene is an interesting candidate whose expression is reduced in colon, pancreas, ovary and oral cancers. The present study investigates the expression and functional effects of *MTUS1* gene products in breast cancer.

**Methods and Findings:**

By means of gene array analysis, real-time RT-PCR and immunohistochemistry, we show here that MTUS1/ATIP3 is significantly down-regulated in a series of 151 infiltrating breast cancer carcinomas as compared to normal breast tissue. Low levels of ATIP3 correlate with high grade of the tumor and the occurrence of distant metastasis. ATIP3 levels are also significantly reduced in triple negative (ER- PR- HER2-) breast carcinomas, a subgroup of highly proliferative tumors with poor outcome and no available targeted therapy. Functional studies indicate that silencing ATIP3 expression by siRNA increases breast cancer cell proliferation. Conversely, restoring endogenous levels of ATIP3 expression leads to reduced cancer cell proliferation, clonogenicity, anchorage-independent growth, and reduces the incidence and size of xenografts grown *in vivo*. We provide evidence that ATIP3 associates with the microtubule cytoskeleton and localizes at the centrosomes, mitotic spindle and intercellular bridge during cell division. Accordingly, live cell imaging indicates that ATIP3 expression alters the progression of cell division by promoting prolonged metaphase, thereby leading to a reduced number of cells ungergoing active mitosis.

**Conclusions:**

Our results identify for the first time ATIP3 as a novel microtubule-associated protein whose expression is significantly reduced in highly proliferative breast carcinomas of poor clinical outcome. ATIP3 re-expression limits tumor cell proliferation *in vitro* and *in vivo*, suggesting that this protein may represent a novel useful biomarker and an interesting candidate for future targeted therapies of aggressive breast cancer.

## Introduction

Despite extensive progress in the field of breast cancer, this disease remains the leading cause of death by malignancy in women worldwide. The identification of novel molecular markers associated with poor clinical outcome of the disease is of considerable interest for tumor classification and treatment, and represents an issue of major public health importance.

Over the past decade, traditional prognostic and predictive biomarkers including estrogen receptor (ER), progesterone receptor (PR) and human epidermal growth factor receptor 2 (HER2) have been routinely assessed by immunohistochemistry in newly diagnosed breast cancer [1]. Tumors that are positive for ER or PR are generally treated by hormonal therapy whereas patients with HER2 positive tumors are candidates for targeted anti-HER2 therapies. Tumors exhibiting a triple negative phenotype (negative for ER, PR and HER2) currently have no targeted treatment available and remain of poor prognosis [2].

The development of gene profiling studies has proven particularly powerful in classifying breast tumors into distinct molecular subgroups with different biological characteristics and clinical outcome [3]. Three main “intrinsic” sub-types of tumors (luminal, HER2-like and basal-like) have been distinguished that turned out to be similar to, although not just recapitulating, those defined by clinical practice [4]–[6]. Thus, the luminal group of tumors is characterized by ER/PR expression whereas HER2-like tumors are mainly ER negative and strongly HER2 positive. Basal-like carcinomas constitute a heterogeneous subtype of tumors with agressive clinical behavior, that predominantly lack the expression of surrogate markers ER, PR and HER2 [2].

Recent high-throughput, genome-wide studies have provided an integrative analysis of genomic copy number alterations and transcriptomic profiles of breast cancer [7], [8], that may facilitate the identification of oncogenic and tumor suppressor pathways specifically associated with each breast cancer molecular subtype [9], [10]. A considerable number of genetic elements of interest altered in breast cancer have thus been identified [11]. The challenge now is to functionally validate the products of these genes to determine whether they may be used clinically as novel prognostic or predictive biomarkers of breast tumors with poor outcome.

The mitochondrial tumor suppressor (*MTUS1*) gene localizes at 8p22, a chromosomal region frequently deleted in epithelial cancers including in poor prognosis breast cancer [12]. Previous studies have shown that *MTUS1* expression levels are reduced in cancers from pancreas [13], ovary [14], colon [15] and head-and-neck [16]. A deletion polymorphism spanning a *MTUS1* coding exon [17] has been shown to significantly associate with decreased risk of familial breast cancer [18]. Our group has previously reported that the *MTUS1* gene contains 17 coding exons which can be alternatively spliced to generate three major transcripts designated ATIP1, ATIP3 and ATIP4 [19], [20]. By real-time RT-PCR, we showed that ATIP1 and ATIP3 are widely distributed in normal human tissues, while expression of ATIP4 is restricted to the central nervous system. High evolutionary conservation of ATIP amino-acid sequences suggests a pivotal role for these proteins in cellular homeostasis [20], but to date only ATIP1 has been cloned and characterized. This isoform has been identified as an interacting partner of the angiotensin II AT2 receptor involved in trans-inactivation of the EGF receptor and subsequent inhibition of extracellular regulated ERK kinase activity and cell proliferation [13], [19], [21]. However, the expression and function of ATIP proteins in breast cancer have not yet been explored.

The present study investigates the expression levels and biological effects of *MTUS1* gene products in infiltrating breast cancer. We show that ATIP3 is the major *MTUS1* splice variant whose expression is significantly reduced in invasive breast tumors of high histological grade and triple-negative phenotype. Molecular cloning and functional studies presented here reveal that ATIP3 is a novel mitotic spindle-associated protein that reduces breast cancer cell division *in vitro* and *in vivo*.

## Methods

### Breast tumor samples

Infiltrating ductal primary breast carcinomas were obtained from patients included between in the prospective database initiated in 1981 by the Institut Curie Breast Cancer group. All patients gave original verbal consent on the use of tumor specimens for research purposes. Eleven normal breast specimens were obtained from breast mammoplasty surgery. Total RNA was extracted by the cesium chloride method and hybridized to DNA microarrays (Human Genome, Affymetrix HG-U133 set) as previously described [22]. Microarray data reported here are in accordance with MIAME guidelines and have been partially published elsewhere [22]. All samples were reviewed and classified by the same breast pathologist (AVS). Histologic nuclear grades were scored (I to III) according to Elston and Ellis [23]. Estrogen receptor (ER) and progesterone receptor (PR) expression were determined by immunohistochemistry [24] and were considered negative when less than 10% of the tumor cell nuclei showed staining. HER2 expression was evaluated by immunostaining as described previously [24] and was considered positive when IHC staining was 3+ (uniform, intense membrane staining of more than 30% of invasive tumor cells), as recommended by Wolff et al. [25]. This study was approved by the institutional review boards and ethics committee of the Institut Curie.

### Cell culture, plasmid constructs and transfections

Human cancer cell lines MDA-MB-231, MDA-MB-468, SK-MES, HeLa and HeLa-H2B were maintained at 37°C with 5% CO2 in DMEM 4.5 g/l glucose supplemented with 10% fetal calf serum (FCS). MCF7 breast cancer cells were grown in DMEM/F12 medium with 5% FCS. All cancer cell lines are available from ATCC. HeLa-H2B cells stably expressing the mCherry-histone were kindly provided by Dr. Valérie Doye (Institut Jacques Monod, Paris). The source, tumor type and histologic characteristics of breast cancer cell lines used in this study have been described in details elsewhere [26], [27].

The coding sequence of human ATIP3a (3812 bp) was obtained by ligating partial 3′ ATIP sequence isolated from human lung cDNA library [19] with the 5′ region of IMAGE clone ID 3835953 (ATCC). Sequencing of the complete cDNA confirmed its identity with Genbank accession number NM_001001924. The full-length ATIP3 sequence was then PCR-amplified and subcloned into the BamHI and XhoI restriction sites of the pEGFP-C1 vector (Clontech) so that the initiating codon of ATIP3 is in frame with the carboxy-terminal end of the Green Fluorescent protein (GFP).

Transfection of plasmid cDNA into HeLa, HeLa-H2B, MDA-MB-231 or MCF7 cells was performed for 24 h using Lipofectamine Reagent 2000 (Invitrogen) as described by the manufacturer. Small interfering RNA (siRNA) was transfected at 50 nM for 72 h using Lipofectamine reagent 2000. Control siRNA duplex (non targeting pool #1), and specific MTUS1 siRNA#1 (on-target plus smart pool, NM020749) and siRNA#2 (on-target plus siRNA duplex, sens strand : UGG CAG AGG UUU AAG GUU A) were purchased from Dharmacon (Chicago, IL, USA). Silencing efficiency was measured by western blot and real time RT-PCR.

### Real-time RT-PCR

Total RNA was extracted from tumor samples by the cesium chloride protocol [28], and from cancer cell lines using Trizol reagent (Invitrogen) according to the manufacturer's instructions. First-strand cDNAs were synthesized using oligo(dT) primer with Superscript™ reverse transcriptase (Invitrogen) for 60 min at 37°C. cDNA (5 ng) was used for real-time PCR analysis using Fast Start SYBR Green I reagent (Roche) and LightCycler instrument. Each reaction was carried out in duplicate. EEF1G and PP1A cDNAs served as internal controls. Gene-specific primer pairs located in different exons (supplemental Table S1) were designed with Oligo6 software. Results are expressed relative to EEF1G values.

### Immunohistochemistry

Immunohistochemistry was performed on 5 µm sections from formalin-fixed, paraffin-embedded tissue of the breast cancer samples. Heat-mediated antigen retrieval was performed in EDTA buffer pH 9 in water bath for 30 min. Immunostaining was performed on a Dako autostainer using a peroxidase-labeled polymer-based detection system (Envision plus, Dako) and diaminobenzidine as a chromogen. Monoclonal anti-MTUS1 antibodies (Abnova) were diluted 1∶40 and incubated overnight at 4°C. Slides were counterstained with hematoxylin. MCF7 cells transfected with GFP-ATIP3 were used as a positive control and non-transfected MCF7 cells were considered as negative control. MTUS1 immunoreactivity in tissue sections was scored based on the percentage of positive cells and the intensity (1 to 3) of the staining. Tumors were considered MTUS1-positive when more than 60% of the cells showed intense (2–3) staining, and were considered MTUS1-negative when less than 30% of the cells showed weak (0–1) immunostaining.

For analysis of xenografts, 3 µm sections were cut from formalin-fixed, paraffin-embedded tissue blocks, then counterstained with hematoxylin-eosin and examined under an inverted microscope.

### Cell proliferation assays

Cells were seeded in quadruplicate in 96-well plates at the density of 5.10^3^ cells per well. Cell proliferation was analyzed by incubation for 4 h at 37°C with 1 mg/ml tetrazolium salt MTT (Sigma). Cleavage products (formazan) were solubilized with DMSO and optical density was measured at 560 nm, as recommended by the manufacturer. DNA synthesis was measured using a colorimetric immunoassay (Roche) after incorporation of 5-bromodeoxyuridine (BrdU) for 4 h at 37°C. Incorporated BrdU was quantified by optical density reading at 405 nm according to manufacturer's instructions.

For clonogenicity experiments, MCF7 cells were transfected for 24 h with 0.2 µg pEGFP-C1 vector or 2 µg of GFP-ATIP3 cDNA. Similar transfection efficiency (50%) was assessed by FACS analysis and cell viability was verified using trypan blue. Cells were plated at various dilutions in 6 well plates and transfectants were selected by adding geneticin (G418, 1 mg/ml, Gibco) in fresh medium twice a week for 3 weeks. Resistant colonies were stained with 0.5% cristal violet (Sigma) and counted.

For anchorage-independent growth assays, stable transfectants were seeded onto 6-well plates on 0.4% agar gel in appropriate medium supplemented with 20% FCS, over a bottom layer of 0.6% low-melting temperature agar gel. Cells were grown for 4 weeks and the formed colonies were counted under an inverted microscope.

### In vivo tumorigenicity assay

Five weeks old female immunodeficient CB17/scid mice were purchased from Harlan (UK). A slow-release 17-beta-estradiol pellet (60-day release, 0.72 mg per pellet, Innovative Research of America, Florida, USA) was implanted subcutaneously into the intrascapular region of each mouse three days before breast cancer cell inoculation. MCF7 cell clones (5×10^6^ cells in 100 µl PBS) were injected subcutaneously into both flanks. All procedures were performed in accordance with institutional guidelines established by the Ministère de l'Agriculture et de la Forêt, Direction des services vétérinaires (Paris, France). Tumor diameter was measured twice weekly using a calipper along two orthogonal axes: length (*L*) and width (*W*). The volume (*V*) of tumors was estimated by the formula *V* = *L*×(*W* ^2^)/2. Mice were sacrificed on day 38 and tumors were excised, weighed and photographed. Tumors were fixed in 4% formaldehyde solution and paraffin-embedded for histologic analysis.

### Immunofluorescence microscopy and live cell imaging

Exponentially growing cells plated on coverslips were fixed in ice-cold methanol for 5 min prior to incubation for 1 hr at room temperature with primary antibodies. SK-MES cells were incubated with human anti-alpha-tubulin antibodies clone F2C diluted 1∶10 [29] and mouse anti-MTUS1 monoclonal antibodies diluted 1∶10 (Abnova, clone 1C7). HeLa, MDA-MB-231 or MCF7 cells transiently transfected (24 hrs) with pEGFP-C1 or GFP-ATIP3 were incubated with mouse anti-alpha-tubulin monoclonal antibodies (Sigma) diluted 1∶500. After extensive washing, cells were incubated for 30 min at room temperature with appropriate secondary Cy-2-conjugated anti-human and/or Cy-3-conjugated anti-mouse antibodies (Jackson Laboratories, Interchim, France) diluted 1∶500. Coverslips were mounted on glass slides using Glycergel Mounting Medium (DakoCytomation) and examined with a Leica TCS SP2 AOBS confocal microscope using appropriate filters. Image analysis was performed with NIH ImageJ software.

For live cell imaging, images were acquired on a spinning disk microscope (one image taken every 5 min for 36 hrs following transient transfection of HeLa-mCherryH2B cells) as described [30]. Multi-dimensional acquisitions were performed in time-lapse mode using Metamorph 7.1.7 software.

### Microtubules binding in cell extract

Microtubule cosedimentation assay was performed as described [31] with modifications. Cells were incubated for 20 min at 4°C in PEM buffer (100 mM PIPES, pH 6.9, 1 mM MgSO4, 1 mM EGTA), scraped and sonicated prior to centrifugation at 15000 rpm for 10 min, 4°C. Clarified samples were incubated with taxol (20 µM) in the presence of GTP (1 mM) and DTT (1 mM) for 45 min at 37°C and were spun at 70 000 g for 30 min at 30°C through a cushion buffer containing 40% glycerol, 20 µM taxol and 1 mM GTP. The supernatant (S) and pellet (P) fractions were collected separately and subsequently immunoblotted with anti-GFP (Sigma) or anti-MTUS1 (Abnova) antibodies. Blots were reprobed using anti-alpha-tubulin (Sigma) antibodies.

### Statistical Analysis

In all studies, values are expressed as mean ± standard deviation (SD). Statistical analyses were performed by unpaired Student's t test and Tukey-Kramer's test using JMP7 software. Differences were considered statistically significant at p<0.05.

## Results

### *MTUS1* gene expression is reduced in invasive breast cancer samples

**Figure 1:**
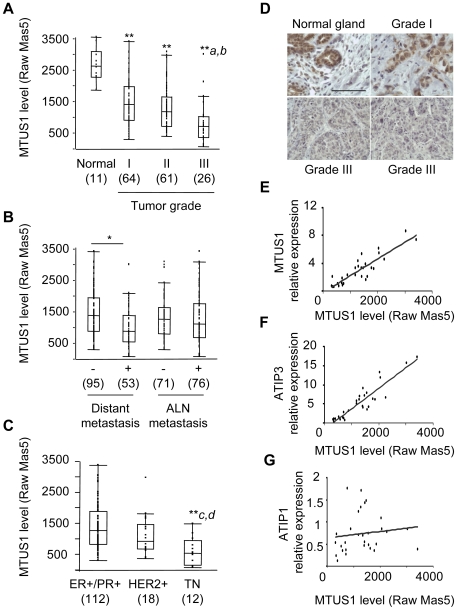
ATIP3 down-regulation in invasive breast carcinomas. A–B. Comparison of MTUS1 (U133A Affymetrix 212096_s_at) probeset data intensities in (A) normal breast tissue and 151 invasive breast tumors classified according to histological grade (I, II, III), (B) breast tumors classified according to the occurrence of distant metastasis or axillary lymph node (ALN) metastasis and (C) molecular subgroups of breast tumors defined by immunodetection of surrogate markers ER/PR+ (luminal), HER2+ (HER2−like) and ER−, PR−, HER2− (triple negative, TN). Probeset intensities were calculated using Affymetrix Raw MAS5.0 default settings. The number of samples is indicated below in brackets. ^*^p<0.001; ^**^p<0.0001 compared to normal tissue; ^a^p<0.0001 compared to grade I; ^b^p<0.005 compared to grade II; ^c^p<0.0001 compared to ER+/PR+; ^d^p<0.05 compared to HER2+. D. Immunohistochemistry on a breast tumor section of histological grade I and adjacent normal breast tissue (upper panel), and two representative grade III breast cancer sections (lower panel) using anti-MTUS1 monoclonal antibodies. A bar represents 100 µm. E–G. Correlation between MTUS1 (212096_s_at) probeset intensities and real-time RT-PCR expressed relative to internal control EEF1G in 29 representative breast tumor samples. Oligonucleotides were designed to amplify total ATIP transcripts (“MTUS1”) (E), ATIP3 transcripts (F), or ATIP1 transcripts (G).

The expression of *MTUS1* transcripts was analyzed in a series of 151 infiltrating ductal breast carcinomas and eleven normal breast tissues. The intensities of each of three specific *MTUS1* probesets (212096_s_at; 212093_s_at; 239576_at) were obtained from our previous U133A Affymetrix DNA array study [22] and compared to histologic characteristics of the tumors and clinical data for the patients (supplemental Table S2). As shown in Fig. 1A and Table 1, *MTUS1* expression levels were significantly reduced in breast cancer samples as compared to normal tissue. *MTUS1* levels were significantly lower in high grade tumors (grade III) as compared to grades I (p<0.0001) and II (p = 0.0032), but did not differ significantly between tumors of grades I and II (p = 0.06). Similar results were obtained with all three *MTUS1* probesets (Fig. 1A and supplemental Fig.S1). As presented in Table 1, a two- to ten-fold reduction in *MTUS1* expression was observed in 47.7% of the tumors, and the number of samples underexpressing *MTUS1* increased with the histological grade : 38.8%, 47.5% and 84.6% in grade I, II and III, respectively. Low levels of *MTUS1* also significantly correlated with tumors that developed metastasis at distant sites, but not with the occurrence of axillary lymph node metastasis (Fig. 1B). *MTUS1* levels were then compared among molecular breast tumors classified clinically according to immunohistochemical detection of the surrogate markers ER, PR and HER2. As shown in Fig. 1C, *MTUS1* Affymetrix probeset intensities were significantly lower in a majority (83.3%) of triple negative (ER−/PR−/HER2−) carcinomas as compared to luminal (ER+) and HER2+ tumor subgroups (Table 1), which supports the conclusion that reduced levels of *MTUS1* are associated with breast cancer aggressive subtypes.

**Table 1:** MTUS1 expression levels in invasive breast carcinomas.

	Number of tumors	Raw Mas5 Median	Raw Mas5 [range]	Raw Mas5 Mean+/−SD	% underexpressed	Number of tumors underexpressed
**Normal**	**11**	2508	[1842.9–3388.6]	2561.5+/−468.8		
**Grade I**	**64**	1563	[532.2–3414.9]	1646.7+/−651.5	**38.8**	21
**Grade II**	**61**	1354	[636.4–3105.5]	1438+/−595.6	**47.5**	29
**Grade III**	**26**	912	[321.5–3030.7]	1005.3+/−616.3	**84.6**	22
**ER+/PR+**	**112**	1443	[616.8–3414.9]	1539.3+/−640.5	**42.8**	48
**HER2+**	**18**	1198.7	[599.6–3030.7	1320.3+/−597.4	**55.5**	10
**TN**	**12**	818.5	[321.5–1635.4]	818.5+/−416.4	**83.3**	10
**All tumors**	**151**	1353.7	[321.5–3414.9]	1452+/−659	**47.7**	72

Raw Mas5 Affymetrix values (probeset 212096_s_at) in cancer samples classified according to their histological grade (I, II, III) and molecular subtypes ER+/PR+, HER2+, TN (triple negative). Tumors with Affymetrix values being more than two-fold lower than those in normal samples were considered underexpressed.

Immunohistochemical analyses were undertaken to examine the cellular distribution and expression of MTUS1 at the protein level *in situ*. Immunohistochemistry performed on normal breast tissue sections using monoclonal anti-MTUS1 antibodies revealed a strong cytosolic staining in luminal epithelial cells of the breast (Fig. 1D). The same results were obtained using polyclonal anti-ATIP antibodies described previously [19] (data not shown). MTUS1 immunostaining was then analyzed in 20 breast cancer samples for which paraffin-embedded tissue sections were available (Fig. 1D). Selected tumors were representative of all histological grades (10, 3 and 7 tumors of grade I, II and III, respectively), and main molecular subtypes (12 ER+, 3 HER2+ and 3 triple negative tumors). MTUS1 immunostaining in tumor sections was scored and compared to *MTUS1* mRNA expression levels according to Affymetrix probeset intensities. As presented in supplemental Table S3, tumors showing low levels of *MTUS1* transcripts by DNA array and/or real-time RT-PCR analysis also displayed undetectable or weak MTUS1 staining, whereas those expressing high levels of *MTUS1* mRNA were attributed a high score by immunohistochemistry. Thus, results on protein expression paralleled gene array analysis.

Results obtained by DNA microarray were further confirmed and extended by real-time RT-PCR. We selected a panel of 29 representative tumor samples whose *MTUS1* Affymetrix probesets intensities ranged from low (n = 11), moderate (n = 13) to high (n = 5). Expression levels of total *MTUS1* transcripts, measured by quantitative PCR using oligonucleotides located in the 3′ exons shared by all ATIP members, were found to significantly correlate (r2 = 0.77) with Affymetrix probeset intensities (Fig. 1E). In order to discriminate between different *MTUS1* spliced variants, real-time RT-PCR was designed to specifically amplify ATIP1 and ATIP3 mRNAs using oligonucleotides located in their specific 5′ exons, respectively. Since ATIP4 transcripts are exclusively expressed in the central nervous system [20], these were not included in the present study. Significant correlation was observed between Affymetrix probeset intensities and expression levels of ATIP3 (r2 = 0.83) (Fig. 1F), but not ATIP1 transcripts (r2 = 0.01) (Fig. 1G), indicating that ATIP3 is the major *MTUS1* splice variant whose expression is regulated in breast cancer.

### ATIP3 inhibits breast cancer cell proliferation

As a pre-requisite to functional studies, a panel of human cancer cell lines was analyzed for *MTUS1* expression by real-time RT-PCR (Supplemental Fig. S2A). Nine out of 13 breast cancer cell lines including MDA-MB-231, MCF7, Hs578T, BT549, MDA-MB-453, T-47D, ZR-75.1, MDA-MB-361 and SK-BR3 showed low to undetectable levels of *MTUS1*, whereas CAMA-1, ZR-75.30, BT-474 and MDA-MB-468 expressed moderate levels of *MTUS1* transcripts. Highest levels of *MTUS1* expression were found in the lung cancer cell line SK-MES, that was therefore selected for further study. As for primary tumors, ATIP3 expression in cancer cells paralleled that of total MTUS1 mRNA, while ATIP1 remained undetectable in every cell line examined. In agreement with our RT-PCR studies, western blot experiments using anti-MTUS1 antibodies revealed a polypeptide of 170 KDa corresponding to ATIP3 in cancer cell lines MDA-MB-468 and SK-MES, but not in MCF7 nor MDA-MB-231 (supplemental Fig.S2B).

**Figure 2:**
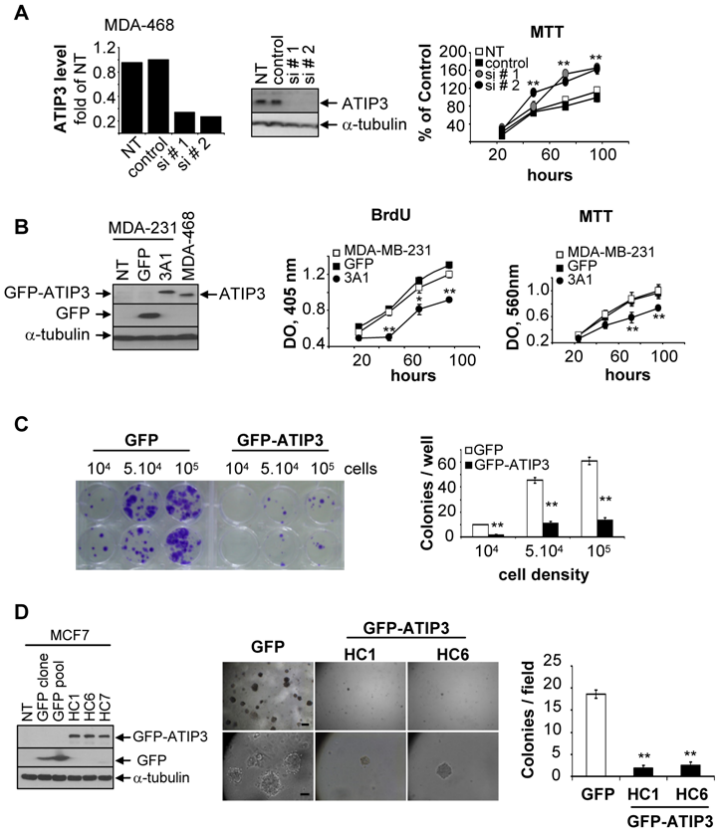
ATIP3 inhibits cancer cell proliferation. A. ATIP3 silencing in MDA-MB-468 cells transfected with control siRNA or specific ATIP3 siRNA#1 or siRNA#2 for 72 hours. *Left panel*: real-time RT-PCR using ATIP3-specific primers relative to EEF1G, normalized to non transfected cells (NT). *Middle panel*: immunoblotting with anti-MTUS1 antibodies showing ATIP3 at 170 kDa, and reprobing with anti-alpha-tubulin antibodies for internal control. *Right panel*: MTT assay. Results are expressed as percent of MTT incorporation in control siRNA-transfected cells at time 96 hours. Shown are the results of two representative experiments out of four performed in quadruplicate. **p<0.0001. B. Cell proliferation in stable MDA-MB-231 transfectants. *Left panel* : immunoblotting of total cell lysates from MDA-MB-231 cells non transfected (NT) or stably expressing GFP or GFP-ATIP3 (clone 3A1) at levels comparable to those in MDA-MB-468. Blots were probed with anti-MTUS1 (upper panel), anti-GFP (middle panel), anti-alpha-tubulin antibodies (lower panel). Arrows indicate the migration of GFP-ATIP3 (200 kDa), ATIP3 (170 kDa), GFP (25 kDa), alpha-tubulin (50 kDa). *Middle and right panels*: measurement of BrdU incorporation and MTT assay. Shown is one representative experiment out of three performed in quadruplicate. **p<0.0001; *p<0.001. C. Colony formation of GFP- or GFP-ATIP3-transfected MCF7 cells plated at different densities. Shown is one representative experiment out of five performed in duplicate. *Right panel* : quantification of the number of colonies per well. **p<0.0001. D. Immunoblotting of total lysates from MCF7 cells left untransfected (NT) or stably transfected with GFP or GFP-ATIP3 (clones HC1 and HC6), revealed as in (B). *Middle panel* : MCF7 stable transfectants grown in soft agar for four weeks. Shown are photomicrographs of one representative experiment out of three performed in triplicate. A bar represents 50 µm (upper panel) and 10 µm (lower panel). *Right panel* : quantification of the number of colonies per field counted under an inverted microscope. **p<0.0001.

The functional consequence of ATIP3 underexpression was investigated by transfection of specific siRNA into the ATIP3-positive MDA-MB-468 cell line. Two different sequences of siRNA successfully silenced ATIP3 expression in MDA-M468 cells both at the mRNA and protein level (Fig. 2A, left panels). As shown in Fig. 2A (right panel), ATIP3 knock-down by both siRNAs led to a significant increase (66.4±5.3% and 63±10.2% upon transfection of siRNA#1 and siRNA#2, respectively) in MDA-MB-468 breast cancer cell proliferation, pointing to an anti-proliferative effect of ATIP3.

To further confirm the inhibitory effects of ATIP3 on breast cancer cell proliferation, a GFP-ATIP3 fusion protein was generated and expressed in ATIP3-negative cells MDA-MB-231 and MCF7. Independent stable transfectants (clone 3A1 in MDA-MB-231, and clones HC1, HC6, HC7 in MCF7) expressed GFP-ATIP3 at levels similar to those of endogenous ATIP3 in MDA-MB-468 (Fig. 2B and 2D, left panel). As shown in Fig. 2B (right panels), cell proliferation measured by BrdU incorporation and MTT assay was reduced by 28±2.3% and 30±5%, respectively, in GFP-ATIP3-transfected clone 3A1 as compared to non transfected and GFP-expressing MDA-MB-231 cells. Similar results were obtained using stably transfected MCF7 clones HC1, HC6 and HC7 (data not shown).

Clonogenicity experiments were then conducted to evaluate the consequences of ATIP3 expression on cell-cell contact growth inhibition. As shown in Fig. 2C, the number of colonies resistant to neomycin were reduced by 85% upon transfection of MCF7 cells with GFP-ATIP3 as compared to GFP.

To investigate the effects of ATIP3 on anchorage-independent growth, which is a hallmark of tumorigenic properties *in vitro*, stably transfected breast cancer cells were allowed to grow for four weeks in soft agar. As shown in Fig. 2D, the number of colonies grown in soft agar were reduced by 75% in MCF7 cell clones transfected with GFP-ATIP3 (HC1 and HC6) as compared to GFP. Similar results were obtained using stably transfected cell clones HC7 and 3A1 (data not shown).

### ATIP3 expression reduces tumor growth *in vivo*

**Figure 3:**
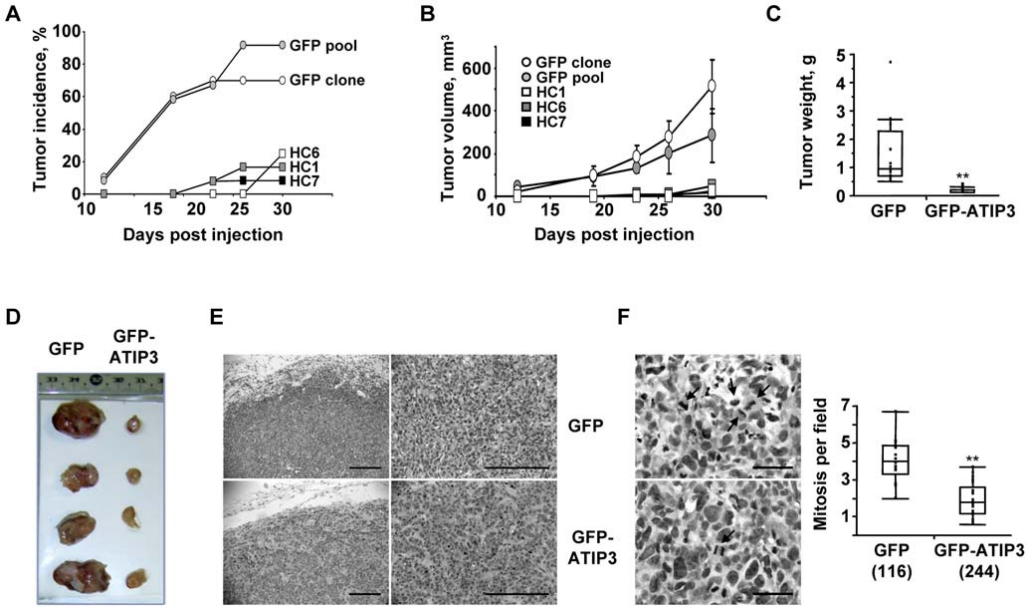
ATIP3 suppresses tumor growth *in vivo*. A. Number of tumor xenografts (in percent) developing after s.c. injection of GFP-ATIP3 expressing MCF7 clones (HC1, HC6, HC7) or GFP-transfected MCF7 cells (stable clone or pool) into immunodeficient mice. B. Time-course of tumor progression in mice injected with GFP or GFP-ATIP3 cell clones as in A. Tumor size was measured twice weekly as indicated in the methods. C. Mean weight (in grams) of tumors excised on day 38 after injection of MCF7 cells expressing GFP (clone and pool, n = 18) and GFP-ATIP3 (clones HC1, HC6, HC7, n = 11). D. Pictures of representative tumors excised on day 38 after injection of MCF7 cell clones as in C. E. Representative histologic analysis of tumors grown from GFP and GFP-ATIP3 expressing MCF7 cells, after hematoxylin-eosin staining. A bar represents 100 µm. F. Representative photomicrographs of cancer cells as in E, stained by hematoxylin-eosin (magnification×63). Arrows show cancer cells undergoing active mitosis. A bar represents 20 µm. Histogram shown on the right is a quantification of the number of mitotic cells per field counted under an inverted microscope. The number of fields analyzed is indicated below in brackets. **p<0.0001.

Stably transfected MCF7 clones (HC1, HC6 and HC7, or GFP) were injected subcutaneously into both flanks of immunodeficient mice, and tumor growth was monitored twice a week. At day 30, only 17% (6/36) of tumor xenografts developed in mice injected with GFP-ATIP3-transfected clones (HC1, HC6 or HC7), as compared with 82% (18/22) in mice injected with GFP-transfected MCF7 cells (Fig. 3A). The time-course of tumor progression (Fig. 3B) and size of the tumors (Fig. 3C, 3D) were significantly reduced in mice injected with HC1, HC6 and HC7 as compared to GFP-expressing MCF7 cells. Histological examination of the tumor nodules excised on day 38 confirmed the presence of tumor cells in each case (Fig. 3E). However a two-fold reduction in the number of cancer cells undergoing active division, assessed by microscopic examination of mitotic cells (Fig. 3F), was observed in tumors expressing GFP-ATIP3 as compared to GFP alone. Thus, restoring endogenous levels of ATIP3 expression leads to reduced breast cancer cell proliferation and delayed tumor growth *in vivo*.

### ATIP3 associates with microtubules

**Figure 4:**
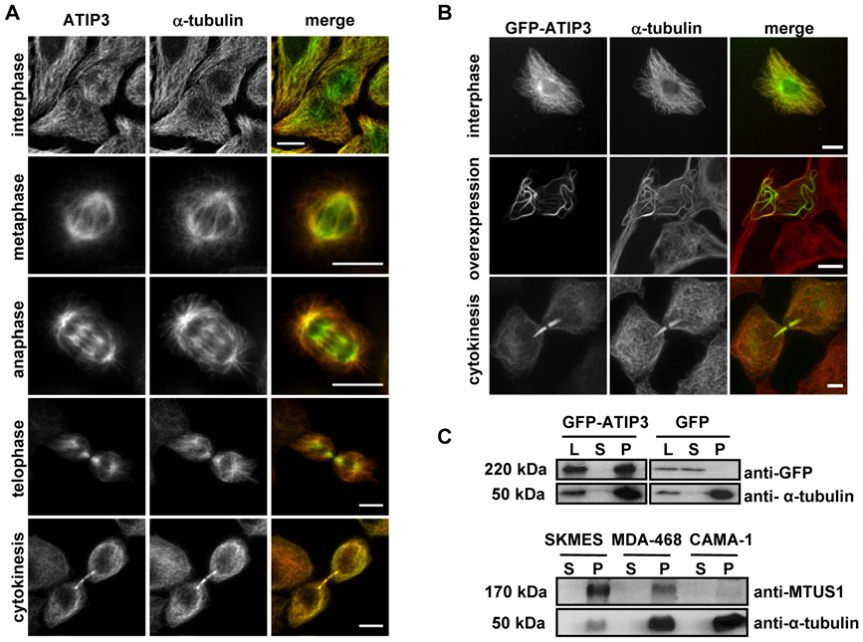
ATIP3 associates with microtubules. A–B. Representative photomicrographs of confocal microscopy analysis showing the cellular distribution of (A) endogenous ATIP proteins in SK-MES cancer cells and (B) transiently transfected (24 h) GFP-ATIP3 fusion protein, stained with anti-MTUS1 (green) and anti-alpha-tubulin (red) antibodies. A bar represents 10 µm. C. Microtubule co-sedimentation assay performed on MDA-MB-231 stable transfectants expressing GFP-ATIP3 or GFP (upper panel) and SK-MES, MDA-MB-468 and CAMA-1 tumor cells expressing endogenous ATIP3 (lower panel). Immunoblots were revealed using anti-GFP or anti-MTUS1 antibodies, and reprobed with anti-alpha-tubulin antibodies. Molecular weights are indicated on the left. L: total cell lysate; S: supernatant; P: pellet.

To further characterize ATIP3 at the molecular level, its subcellular localization was analyzed in SK-MES cancer cells that express high levels of ATIP3 and no detectable ATIP1. Confocal microscopy analyses (Fig. 4A) allowed the detection of endogenous ATIP3 proteins as filamentous structures in the cytoplasm, with a staining pattern similar to that of microtubules. Incubation with anti-alpha-tubulin antibodies revealed colocalization of ATIP3 with the microtubule network during interphase. During mitosis, endogenous ATIP3 staining decorates the mitotic spindle in metaphase and anaphase, and remains strongly associated with the intercellular bridge during cytokinesis (Fig. 4A). To confirm that the ATIP3 isoform indeed colocalizes with microtubules, human cell lines were transiently transfected with the GFP-ATIP3 fusion protein and analyzed by fluorescence microscopy. As shown in Fig. 4B, GFP-ATIP3 colocalizes with alpha-tubulin at the microtubule cytoskeleton and microtubule organizing center (MTOC) in cells expressing low levels of the fusion protein. Higher levels of expression of the GFP-ATIP3 construct led to the formation of bundles which is a characteristic feature of stabilized microtubules, suggesting that ATIP3 may regulate the dynamics of microtubule polymerization. The same intracellular localization was observed upon transfection of GFP-ATIP3 into HeLa, RPE1, MDA-MB-231 and MCF7 cells (not shown). As observed for endogenous ATIP3, the GFP-ATIP3 fusion protein also localizes at the mitotic spindle during mitosis (not shown), and stains the intercellular bridge during cytokinesis (Fig. 4B).

Microtubule co-sedimentation assays were then performed on cells transfected with either GFP-ATIP3 or GFP. In this assay, microtubules are stabilized in the presence of taxol and GTP, and proteins that associate with microtubules are captured in the pellet fraction. As shown in Fig. 4C (upper panel), the whole pool of GFP-ATIP3 co-sediments with stabilized microtubules in the pellet whereas the soluble GFP protein is only detected in the supernatant fraction. Co-sedimentation of GFP-ATIP3 with microtubules was observed in both MCF7 and MDA-MB-231 cells either stably or transiently transfected with GFP-ATIP3 (data not shown).

Whether endogenous ATIP3 also co-sediments with microtubules was further evaluated in SK-MES, MDA-MB-468 and CAMA-1 cancer cells, that express high to moderate levels of ATIP3 (supplemental Fig.S2). As shown in Fig. 4C (lower panel), the whole pool of endogenous ATIP3 was detected in the microtubule pellet and no residual soluble protein was found in the supernatant. Thus, ATIP3 associates with stabilized microtubules.

### ATIP3 promotes prolonged mitosis

**Figure 5:**
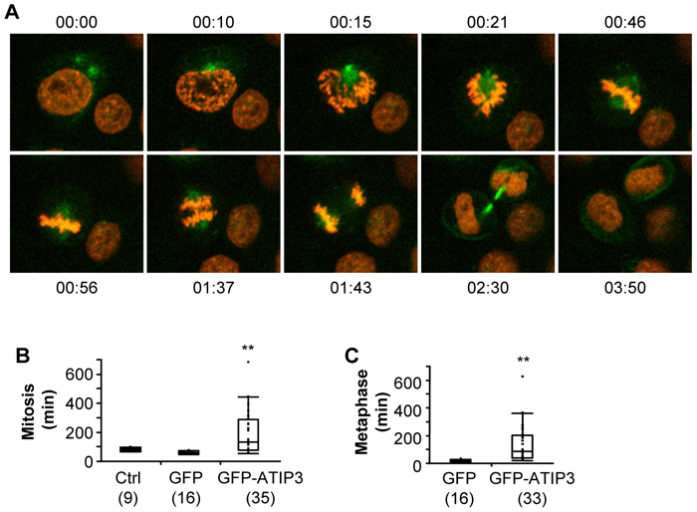
ATIP3 delays mitosis. A. Representative photomicrographs from live cell imaging of HeLa cells stably expressing mCherry-histone 2B (in red) and transiently transfected with GFP-ATIP3 (in green). Time (in hrs) is indicated above or below each panel. B. Time (in min) necessary to achieve mitosis in control, GFP-transfected and GFP-ATIP3 transfected cells. The number of cells analyzed is indicated below in brackets. **p<0.0001. C. Time (in min) between chromosome condensation and the onset of anaphase in GFP-transfected and GFP-ATIP3 transfected cells. The number of cells analyzed is indicated below in brackets. **p<0.0001.

The localization of ATIP3 at the mitotic spindle and intercellular bridge, together with its anti-proliferative effects *in vitro* and *in vivo*, prompted us to investigate the effects of this protein in essential steps of cell division. To this end, GFP-ATIP3 was transiently transfected into HeLa-H2B cells, that stably express a red fluorescent protein-tagged Histone H2B and constitute a useful tool to monitor mitosis by live cell imaging. Time-lapse fluorescence videomicroscopy analyses revealed the association of GFP-ATIP3 with duplicated centrosomes during prophase, and further confirmed its localization at the mitotic spindle and intercellular bridge at different steps of mitosis in living cells (Fig. 5A, supplemental video S1 and supplemental video S2). In addition, measuring the time-lapse between chromosome condensation and the end of cytokinesis revealed that cells expressing GFP-ATIP3 need a longer time to achieve mitosis (187±138 min, n = 35) as compared to non-transfected cells (81±12 min, n = 9) and cells expressing GFP (59±7 min, n = 16) (Fig. 5B). More specifically, the time spent in metaphase (from chromosome condensation in prometaphase to the beginning of anaphase) was significantly increased in GFP-ATIP3 (135.9±129.7 min, n = 33) as compared to GFP-transfected cells (19±5.6 min, n = 16) (Fig. 5C). Thus, ATIP3 overexpression delays the progression of mitosis by prolonging the step of metaphase.

## Discussion

The results presented here identify ATIP3 as a novel anti-mitotic protein whose expression is reduced in infiltrating breast cancer. Low levels of expression of ATIP3 are associated with high histological grade of the tumor and the occurrence of distant metastasis, indicating a close relationship between reduced ATIP3 expression and breast cancer aggressiveness. In addition, in tumors showing a triple-negative phenotype (ER− PR− HER2−) ATIP3 levels are significantly lower than those measured in luminal (ER+) and HER2+, suggesting that ATIP3 may represent a novel molecular marker for poor outcome of breast cancer.

Immunohistochemical analyses revealed a cytosolic ATIP localization in luminal epithelial cells of normal breast tissue. ATIP protein immunostaining intensity paralleled its mRNA levels determined both by gene microarray and real-time RT-PCR. Thus, immunodetection of ATIP proteins in breast cancer samples may be a simple and useful tool for pathologists to distinguish between ATIP-positive and negative tumors. This may be of particular interest for the identification of a subset of triple negative breast tumors having lost the expression of ATIP3. Indeed, in contrast to the ER+ and HER2+ tumors subtypes that can be efficiently treated with hormonal and anti-HER2 therapy, respectively, triple negative tumors lack targeted treatments. We show here that restoring ATIP3 expression in ATIP3-negative breast cancer cell lines leads to reduced cancer cell proliferation, clonogenicity and anchorage-independent growth. Furthermore, ATIP3 re-expression lowers the incidence, time-course and size of tumor progression in xenograft models *in vivo*, suggesting that ATIP3 may represent an interesting candidate for future targeted therapies of triple negative breast tumors. Further studies will be necessary to investigate the feasibility of *in vivo* methods aimed at re-expressing ATIP3, or active truncated domains of the protein, by using tumor-targeted systemic delivery of nanocomplexes as recently reported for the tumor suppressor RB94 [32].

ATIP3 belongs to a family of proteins (ATIP1 to ATIP4) encoded by alternative splicing of *MTUS1*, a candidate 8p22 tumor suppressor gene [20]. Previous studies have reported that *MTUS1* expression levels are reduced in cancers from pancreas, colon, ovary and head-and-neck [13]–[16]. However, the question of which *MTUS1* transcript is down-regulated had not been investigated, nor had the *in vivo* consequence of ATIP re-expression been analyzed until now. We report here for the first time that ATIP3 is the major *MTUS1* isoform whose expression is altered in invasive breast cancer. Our results showing that ATIP3 re-expression functionally reduces breast cancer cell growth *in vitro* and *in vivo* may argue in favor of a tumor suppressor effect of ATIP3. However, whether *MTUS1* corresponds to a “bona fide” tumor suppressor gene inactivated by epigenetic or genomic alterations remains to be established. Of interest, our previous mutational analysis of *MTUS1* in a series of hepatocellular carcinomas and cell lines has identified five potential gene mutations which are all located in exons specific to the ATIP3 transcript [33]. We and others have also identified *MTUS1* gene variations located on ATIP3-specific exons in head-and-neck carcinoma cell lines [33], [16]. Furthermore, Frank et al. have recently reported significant association between copy number variants of MTUS1 exon 4 - specific to the ATIP3 transcript - and familial breast cancer risk [18], further suggesting that alterations of the ATIP3 isoform may be associated with carcinogenesis. To note, the functional consequences of *MTUS1* gene alterations could not be examined until now due to a lack of knowledge on ATIP3 intracellular actions. By providing a molecular and functional analysis of ATIP3, the present study brings a major contribution to the characterization of *MTUS1* gene products and to the identification of potential driver mutations that may lead to a loss of function of the ATIP3 protein in cancer.

Our results show that ATIP3 is a novel microtubule-associated protein localized at the centrosome, mitotic spindle and intercellular bridge, and that its overexpression delays the progression of mitosis in living cells. ATIP3 thus appears as a new member of a functional family of microtubule-associated proteins, including adenomatous polyposis coli APC [34], LZTS1 [35] and RASSF1A [31], [36], that interfere with the microtubule cytoskeleton to regulate essential steps of mitosis and have been clearly identified as tumor suppressors down-regulated or mutated in breast cancer.

The possibility that ATIP3 may be involved in cell division *via* a regulation of spindle dynamics is consistent with its localization at centrosomes, spindle poles and spindle microtubules, and is supported by its effects on microtubule stability (our unpublished observations). ATIP3 may thus represent a novel interesting target for rational anti-mitotic therapies based on proteins associated with the mitotic spindle [37], [38]. Furthermore, the observation that ATIP3 promotes prolonged mitosis by extending the time of metaphase potentially reflects modulation of spindle checkpoint signalling and provides a molecular basis for elucidating the intracellular functions of ATIP3 in cell division. Thus, the novel microtubule associated protein ATIP3 identified here may represent a useful prognostic biomarker of agressive breast cancer and a promising candidate for the development of new targeted therapeutic strategies.

## Supporting Information

Figure S1 MTUS1 down-regulation in invasive breast carcinomas. U133A Affymetrix MTUS1 probesets (239576_at; 212093_s_at) intensities in normal breast tissue and 151 invasive breast tumors classified according to histological grade (I, II, III). Probeset intensities were calculated using Affymetrix Raw MAS5.0 default settings. The number of samples is indicated below under brackets.
(1.57 MB TIF)

Figure S2 MTUS1/ATIP expression in human cancer cell lines. A. Real-time RT-PCR on 14 human tumor cell lines using MTUS1, ATIP1 or ATIP3 primers as defined in the methods, expressed relative to internal control EEF1G. B. Immunoblotting of total cell lysates from cell lines MCF7, MDA-MB-231, MDA-MB-468 and SKMES using anti-MTUS1 monoclonal antibodies. Blots were reprobed with anti-ezrin for internal control. Arrows on the left indicate apparent molecular weights of endogenous ATIP3 (170 kDa) and ezrin (80 kDa).
(1.46 MB TIF)

Table S1 Sequences and gene location of oligonucleotides used in real-time RT-PCR.
(0.03 MB DOC)

Table S2 MTUS1 affymetrix probesets intensities in 151 invasive breast carcinomas. Intensities of three MTUS1 probesets (212093_s_at; 212096_s_at; 239576_at) in 151 infiltrating ductal carcinomas (IDC) and 11 normal breast tissues. Values (Raw MAS05 setting defaults) are compared to the histological grade (Easton and Ellis, EE) of the tumors and the occurrence of axillary lymph node (ALN) and distant metastasis. Immunohistochemical detection of surrogate markers : epidermal growth factor receptor 1 (EGFR), human epidermal growth factor receptor 2 (HER2), estrogen receptor (ER), and progesterone receptor (PR), is as described in the Methods. EGFR and HER2 values were scored 0 to 100 according to the percentage of stained cells. ND : not determined.
(0.05 MB XLS)

Table S3 Comparison of MTUS1 mRNA levels and protein immunostaining in 20 invasive breast carcinomas. Affymetrix 212096_s_at probeset intensities (Raw MAS5.0) values, total MTUS1 mRNA levels measured by real-time PCR relative to EEF1G internal control, and immunohistochemical (IHC) results using anti-MTUS1 monoclonal antibodies. MTUS1 mmunostaining was scored as described in the Methods, according to the percentage (%) of positive tumor cells and intensity of the staining. Values are compared to histological grade (I, II, III) of the tumor and immunodetection of surrogate markers (ER, PR, EGFR, HER2) determined as described in the Methods. nd : not determined.
(0.01 MB XLS)

Video S1 Live cell imaging of GFP-ATIP3 in HeLa-H2B cells. HeLa-H2B cells stably expressing mcherry-Histone 2B were transfected for 24 hrs with GFP-ATIP3 and imaged by spinning disk confocal microscopy in a single focal plane every five minutes for 36 hrs following transfection. GFP-ATIP3 fluorescence labeling (in green) associates with the centrosomes, microtubule spindle, cleavage furrow and intercellular bridge during mitotic progression of live transfected cells. Fluorescence labeling of DNA is in red.
(3.83 MB MOV)

Video S2 Live cell imaging of GFP-ATIP3 in HeLa-H2B cells. HeLa-H2B cells stably expressing mcherry-Histone 2B were transfected for 24 hrs with GFP-ATIP3 and imaged by spinning disk confocal microscopy in a single focal plane every five minutes for 36 hrs following transfection. GFP-ATIP3 fluorescence labeling (in green) associates with the centrosomes, microtubule spindle, cleavage furrow and intercellular bridge during mitotic progression of live transfected cells. Fluorescence labeling of DNA is in red.
(2.20 MB MOV)
