# Supplementary figures and images for: 8p22 MTUS1 Gene Product ATIP3 Is a Novel Anti-Mitotic Protein Underexpressed in Invasive Breast Carcinoma of Poor Prognosis
Source: PLoS One. 2009 Oct 1;4(10):e7239. doi: 10.1371/journal.pone.0007239 (PMC2749209; doi:10.1371/journal.pone.0007239)

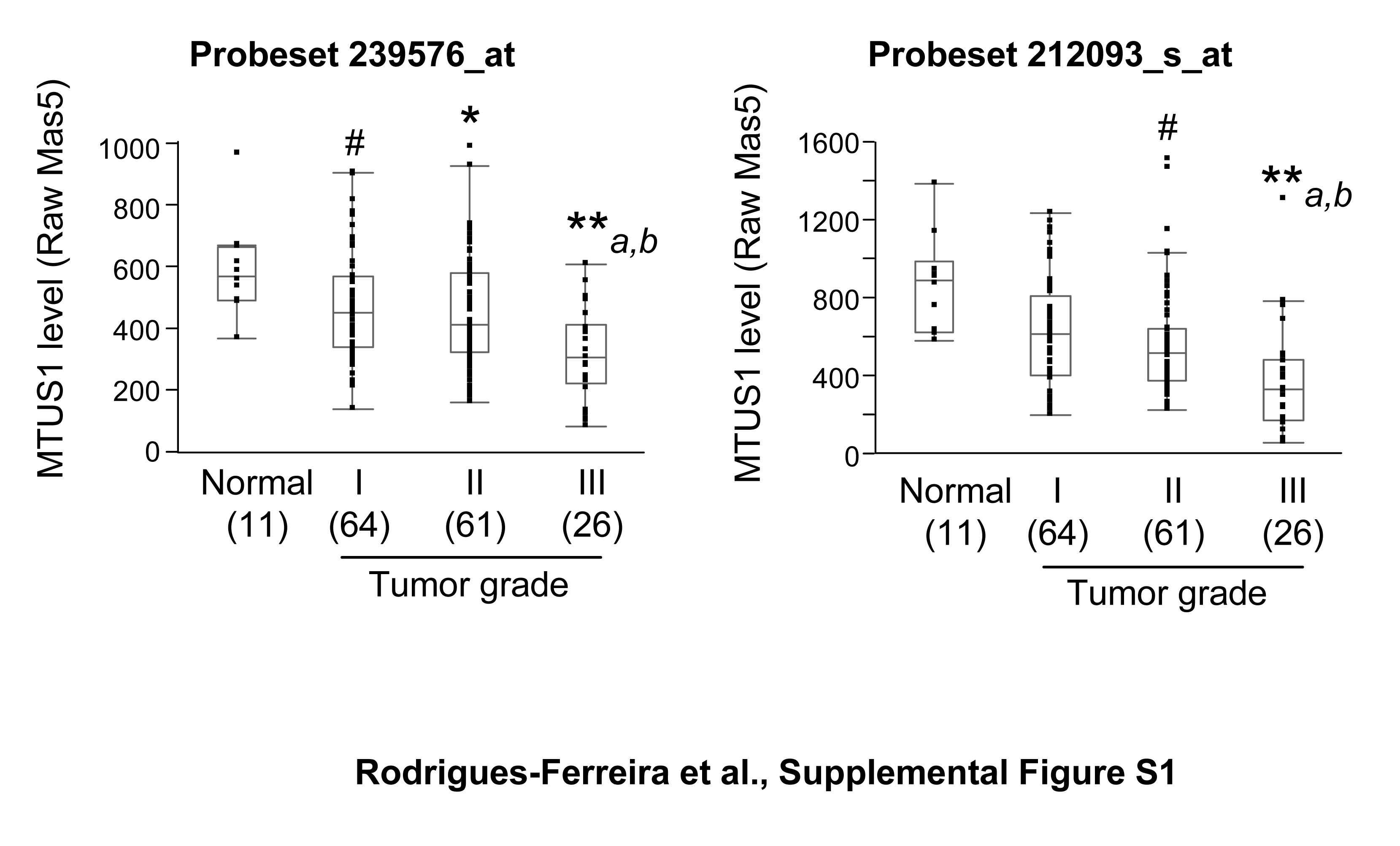

Supplement: Figure S1 — MTUS1 down-regulation in invasive breast carcinomas. U133A Affymetrix MTUS1 probesets (239576_at; 212093_s_at) intensities in normal breast tissue and 151 invasive breast tumors classified according to histological grade (I, II, III). Probeset intensities were calculated using Affymetrix Raw MAS5.0 default settings. The number of samples is indicated below under brackets. (1.57 MB TIF) [file pone.0007239.s001.tif]

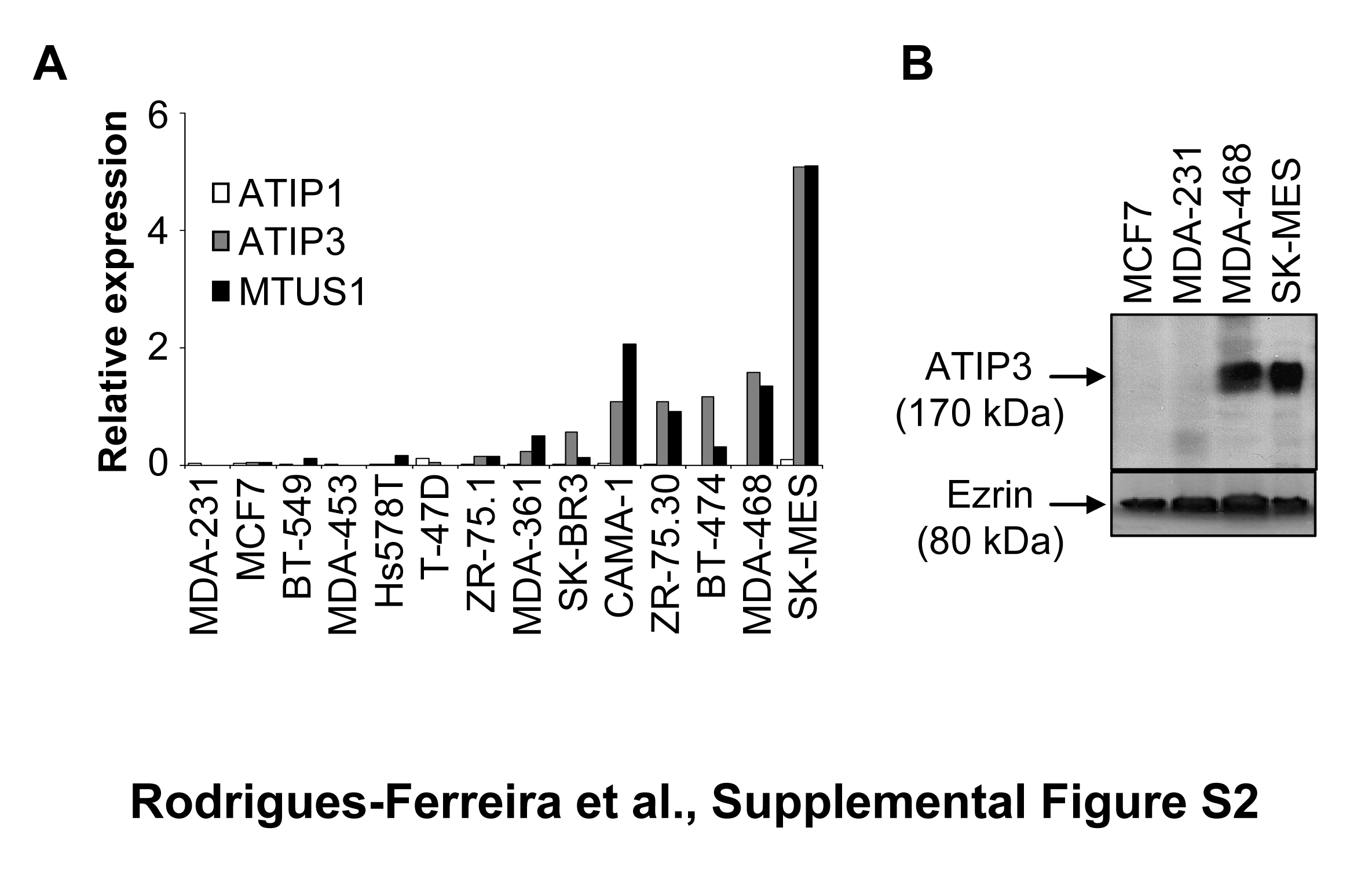

Supplement: Figure S2 — MTUS1/ATIP expression in human cancer cell lines. A. Real-time RT-PCR on 14 human tumor cell lines using MTUS1, ATIP1 or ATIP3 primers as defined in the methods, expressed relative to internal control EEF1G. B. Immunoblotting of total cell lysates from cell lines MCF7, MDA-MB-231, MDA-MB-468 and SKMES using anti-MTUS1 monoclonal antibodies. Blots were reprobed with anti-ezrin for internal control. Arrows on the left indicate apparent molecular weights of endogenous ATIP3 (170 kDa) and ezrin (80 kDa). (1.46 MB TIF) [file pone.0007239.s002.tif]
