# Supplementary material for: 8p22 MTUS1 Gene Product ATIP3 Is a Novel Anti-Mitotic Protein Underexpressed in Invasive Breast Carcinoma of Poor Prognosis
Source: PLoS One. 2009 Oct 1;4(10):e7239. doi: 10.1371/journal.pone.0007239 (PMC2749209; doi:10.1371/journal.pone.0007239)
Supplement: Table S1 — Sequences and gene location of oligonucleotides used in real-time RT-PCR. (0.03 MB DOC) [file pone.0007239.s003.doc]

| Sequence | Forward primer | Exon location | Reverse primer | Exon location | PCR product (bp) | Annealing T°C |
| --- | --- | --- | --- | --- | --- | --- |
| MTUS1 | AGCTTCGGGACACTTACATT | 11 | ATAGGCCTTCTTTAGCAATTC | 12 | 149 | 60 |
| ATIP1 | TTCACCATTCACATACGACT | 8 | TTTGCCTGCTCTTTTCAACTGT | 8-9 | 112 | 57 |
| ATIP3 | GGCGGAACAGTGACAATA | 2 | GCAAATTCACCCATGACGA | 4 | 127 | 57 |
| EEF1G | AGATGGCCCAGTTTGATGCTAA | 6-7 | GCTTCTCTTCCCGTGAACCCT | 7 | 90 | 55 |

**Supplemental Table SI : oligonucleotides used in real-time RT-PCR**
